# Two-color second-order sideband generation in an optomechanical system with a two-level system

**DOI:** 10.1038/s41598-018-19556-y

**Published:** 2018-01-18

**Authors:** Cui Kong, Sha Li, Cai You, Hao Xiong, Ying Wu

**Affiliations:** School of Physics, Huazhong University of Science and Technology, Wuhan, 430074 People’s Republic of China

## Abstract

Second-order sideband generation in an optomechanical system with the coupling between a mechanical resonator and a two-level system is discussed beyond the conventional linearized description of optomechanical interactions. The features of two-color second-order sideband generation are demonstrated in this hybrid system. We discovery that the switch between one- and two-color second-order sideband generation is easily realized by shifting the detuning between the control field and the cavity field or the transition frequency of the two-level system. The efficiency of two-color second-order sideband generation increases monotonously with the control field strength. The coupling strength between the mechanical resonator and the two-level system plays a decisive role in the appearance of the two-color second-order sidebands. The two-color second-order sideband generation may provide measurement with higher precision in new degrees of freedom.

## Introduction

Cavity optomechanics^1–3^, which explores the coupling between the optical cavity and the mechanical oscillation via radiation-pressure, has played an indispensable position in modern physics. The subject of rapid developments brings about significant value in both fundamental physics and practical applications, such as achieving high precision measurement^4–7^, slowing and storage of light pulses^8,9^, information processing^10,11^ and optomechanical induced transparency(OMIT)^12,13^. Micro-mechanical resonators^14^ are in combination with optical cavities, which presents some important  phenomena, such as phonon blockade^15,16^, phonon-induced transparency^17^, and high-harmonic generation^18^.

Recently, due to the nonlinear optomechanical interactions between the optical cavities and the mechanical resonators, a lot of interesting phenomena^19–22^ emerge, such as high-order sidebands^23,24^ and chaos^25,26^ which are achieved in the bichromatic driving regime by making use of numerical calculation method. In the perturbative regime that the probe field is weaker than the control field, an analytical method has also been proposed to describe the nonlinear interactions in an optomechanical system^27–29^. Here we use the perturbation method to study the second-order sideband generation in a hybrid optomechanical system with a two-level system coupled to the mechanical resonator.

The hybrid optomechanical system may consist of an optical cavity, a mechanical resonator and other objects including two-level atoms^30^, superconducting qubit circuits^31^, two-level defects^32^ and so on. There are three situations in a hybrid optomechanical system for the coupling among an optical cavity, a mechanical resonator and another object: a two-level system is coupled to both the cavity field and the mechanical mode^33^; a two-level atom is only coupled to the cavity field^30^ and a two-level system is only coupled to the mechanical resonator^34^. For the third situation, the hybrid optomechanical system has been utilized to complete both the classical and quantal researches, such as the appearance of the two transparent windows^34^ and tunable photon blockade^35^. For other similar systems, the discovery of some new applications in the double-electromagnetic-induced transparency (EIT)^36,37^ and double-OMIT^38,39^ has been presented subsequently. All of these results inspire us to explore second-order upper sideband of double frequencies in output spectrum, which is called two-color second-order sideband generation here.

In the present work, we show the two-color second-order sideband generation in a hybrid optomechanical system where a two-level system is only coupled to the mechanical resonator. The interpretation is given for the formation of two-color second-order sidebands and the features of two-color second-order generation are exhibited. The switch between the one- and two-color second-order sideband generation can be actualized by transforming the transition frequency of the two-level system compared to the mechanical-mode frequency, or the detuning between the cavity field and the control field, whose strength sets a direct influence on the efficiency of the second-order sideband generation. In addition, the coupling strength between the mechanical resonator and the two-level system determines whether two-color second-order sidebands can appear. The two-color second-order sideband generation may find potential applications in optical frequency comb^40^ and provides measurement with higher precision in new degrees of freedom, such as charge measurement^4^ and force sensors^41^.

## Results

### Model, Hamiltonian, steady states, and the second-order sideband generation

**Figure 1:**
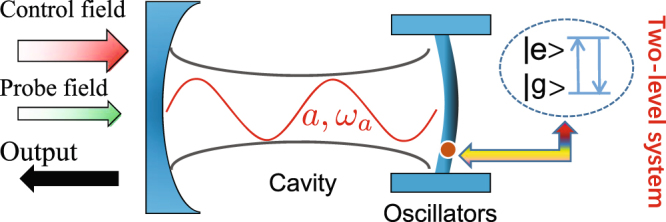
Schematic diagram of an optomechanical system where the mechanical resonator is coupled to a two-level system. The hybird optomechanical system is driven by a strong control field with frequency *ω*_*c*_. When the control field and a weak probe field with frequency *ω*_*p*_ are incident upon the system, the output field is described by *S*_*out*_ with frequencies *ω*_*c*_ ± *n*Δ where n is a positive integer. Through the transformation of the control field strength, the detuning between the cavity field and the control field, and the transition frequency of the two-level system, we can get some novel features of the second-order sidebands.

The hybrid optomechanical system consists of an optical cavity and a mechanical resonator coupled to a two-level system, which might be an intrinsic defect inside the mechanical resonator or a superconducting artificial atom, as shown in Fig. 1. Before considering the input laser fields, the Hamiltonian of the system is given by

1 $$\begin{array}{rcl}{\hat{H}}_{0} & = & \hslash {\omega }_{a}{\hat{a}}^{\dagger }\hat{a}+\hslash {\omega }_{b}{\hat{b}}^{\dagger }\hat{b}+\frac{\hslash }{2}{\omega }_{q}{\hat{\sigma }}_{z}-\hslash \chi {\hat{a}}^{\dagger }\hat{a}({\hat{b}}^{\dagger }+\hat{b})\\  &  & +\hslash g({\hat{b}}^{\dagger }{\hat{\sigma }}_{-}+{\hat{\sigma }}_{+}\hat{b}),\end{array}$$

where $\hat{a}$ $({\hat{a}}^{\dagger })$,$\hat{b}$ $({\hat{b}}^{\dagger })$ are the annihilation (creation) operators of the optical field and the mechanical resonator with frequencies *ω*_*a*_, *ω*_*b*_, respectively. The Pauli operator ${\hat{\sigma }}_{z}$, which is atomic inversion operator, usually describes the two-level system and *ω*_*q*_ is the transition frequency of the two-level system. The coupling parameter between the cavity field and the mechanical resonator, which is coupled to the two-level system with coupling strength *g*, is *χ*. Here we treat the interaction between the single-mode optical field and the mechanical resonator as radiation-pressure Hamiltonian. For simplicity, we assume that the mechanical resonator interacted with the two-level system is regarded as Jaynes-Cummings Hamiltonian, and we have no consideration on the direct interaction between the cavity field and the two-level system. The last term, where ${\hat{\sigma }}_{-}$ and ${\hat{\sigma }}_{+}$ are the ladder operators of the two-level system, omits two parts ${\hat{b}}^{\dagger }{\hat{\sigma }}_{+}$ and $\hat{b}{\hat{\sigma }}_{-}$ by means of rotating wave approximation.

Although the Hamiltonian ${\hat{H}}_{0}$ dose not give the direct coupling between the cavity field and the two-level system, we can get the relational expression $\hslash g\chi {\hat{\sigma }}_{x}{\hat{a}}^{\dagger }\hat{a}/{\omega }_{b}$ via applying a unitary transform *U* = exp$[-\chi {\hat{a}}^{\dagger }\hat{a}({\hat{b}}^{\dagger }-\hat{b})/{\omega }_{b}]$ to Eq. (1). This explains that there are nonlinear interactions between the two-level system and the cavity field.

The hybrid optomechanical system is driven by a strong control field with frequency *ω*_*c*_. When the strong control field and a weak probe field with frequency *ω*_*p*_ are applied to the optomechanical system, the whole Hamiltonian of the hybrid system can be written as

2 $$\hat{H}={\hat{H}}_{0}+i\hslash ({\rm{\Omega }}{e}^{-i{\omega }_{c}t}{\hat{a}}^{\dagger }+{\varepsilon }{e}^{-i{\omega }_{p}t}{\hat{a}}^{\dagger })+H\mathrm{.}C\mathrm{.,}$$

where the parameter Ω is the Rabi frequency of the control field, which describes the coupling strength between the control field and the cavity field, and the parameter *ε* is the Rabi frequency of the probe field, which describes the coupling strength between the probe field and the cavity field. In a frame rotating at *ω*_*c*_, the Hamiltonian of Eq. (2) can be obtained as

3 $$\begin{array}{c}{\hat{H}}_{r}=\hslash {{\rm{\Delta }}}_{a}{\hat{a}}^{\dagger }\hat{a}+\hslash {\omega }_{b}{\hat{b}}^{\dagger }\hat{b}+\frac{\hslash }{2}{\omega }_{q}{\hat{\sigma }}_{z}-\hslash \chi {\hat{a}}^{\dagger }\hat{a}({\hat{b}}^{\dagger }+\hat{b})\\ \quad \quad \,+\hslash g({\hat{b}}^{\dagger }{\hat{\sigma }}_{-}+{\hat{\sigma }}_{+}\hat{b})+i\hslash ({\rm{\Omega }}{\hat{a}}^{\dagger }+\varepsilon {e}^{-i{\rm{\Delta }}t}{\hat{a}}^{\dagger })+H\mathrm{.}C\mathrm{.,}\end{array}$$

with the detunings Δ_*a*_ = *ω*_*a*_−*ω*_*c*_ and Δ = *ω*_*p*_ − *ω*_*c*_.

Considering the damping and environmental noise terms, the Heisenberg-Langevin equations of motion are read as follows:

4 $$\dot{\hat{a}}=-({\gamma }_{a}+i{{\rm{\Delta }}}_{a})\hat{a}+{\rm{\Omega }}+\varepsilon {e}^{-i{\rm{\Delta }}t}+i\chi \hat{a}({\hat{b}}^{\dagger }+\hat{b})+\sqrt{2{\gamma }_{a}}{\hat{a}}_{in}(t),$$

5 $$\dot{\hat{b}}=-({\gamma }_{b}+i{\omega }_{b})\hat{b}+i\chi {\hat{a}}^{\dagger }\hat{a}-ig{\hat{\sigma }}_{-}+\sqrt{2{\gamma }_{b}}{\hat{b}}_{in}(t),$$

6 $${\dot{\hat{\sigma }}}_{-}=-(\frac{{\gamma }_{q}}{2}+i{\omega }_{q}){\hat{\sigma }}_{-}+ig\hat{b}{\hat{\sigma }}_{z}+\sqrt{{\gamma }_{q}}{\hat{{\rm{\Gamma }}}}_{-}(t),$$

7 $${\dot{\hat{\sigma }}}_{z}=-{\gamma }_{q}({\hat{\sigma }}_{z}+\mathrm{1)}-2ig(\hat{b}{\hat{\sigma }}_{+}-{\hat{b}}^{\dagger }{\hat{\sigma }}_{-})+\sqrt{{\gamma }_{q}}{\hat{{\rm{\Gamma }}}}_{z}(t),$$

where *γ*_*a*_, *γ*_*b*_ and *γ*_*q*_ are the corresponding decay rates of the cavity field, mechanical resonator and two-level system. The environment noises of the cavity field, mechanical resonator and two-level system are described by ${\hat{a}}_{in}(t)$, ${\hat{b}}_{in}(t)$ and ${\hat{{\rm{\Gamma }}}}_{i}(t)$(*i* = −, z), respectively.

In this work, we consider the mean response of the hybrid system. By making use of the lowest-order correlation-truncation approximation, the operators in Eqs. (4) to (7) are reduced to their expectation values, viz. $\langle \hat{a}\rangle =a$, $\langle \hat{b}\rangle =b$, $\langle {\hat{\sigma }}_{-}\rangle ={\sigma }_{-}$ and $\langle {\hat{\sigma }}_{z}\rangle ={\sigma }_{z}$. In addition, we drop the environment noises because $\langle {\hat{a}}_{in}(t)\rangle =\langle {\hat{b}}_{in}(t)\rangle =\langle {\hat{{\rm{\Gamma }}}}_{-}(t)\rangle =\langle {\hat{{\rm{\Gamma }}}}_{z}(t)\rangle =0$. The reduced Heisenberg-Langevin equations of motion then become

8 $$\dot{a}=-({\gamma }_{a}+i{{\rm{\Delta }}}_{a})a+{\rm{\Omega }}+\varepsilon {e}^{-i{\rm{\Delta }}t}+i\chi a({b}^{\dagger }+b),$$

9 $$\dot{b}=-({\gamma }_{b}+i{\omega }_{b})b+i\chi {a}^{\dagger }a-ig{\sigma }_{-},$$

10 $${\dot{\sigma }}_{-}=-(\frac{{\gamma }_{q}}{2}+i{\omega }_{q}){\sigma }_{-}+igb{\sigma }_{z},$$

11 $${\dot{\sigma }}_{z}=-{\gamma }_{q}({\sigma }_{z}+\mathrm{1)}-2ig(b{\sigma }_{+}-{b}^{\dagger }{\sigma }_{-}\mathrm{).}$$

We regard the probe field as a perturbation in the steady state provided by the control field on Eqs. (8–11), so the steady-state solutions can be obtained as *M*_0_(*M* = *A*,*B*, *L*,*Z*) which correspond to the values of the *a*, *b*, *σ*_−_, and *σ*_*z*_, respectively.

12 $${t}_{3}{B}_{0}^{3}+{t}_{2}{B}_{0}^{2}+{t}_{1}{B}_{0}+{t}_{0}=\mathrm{0,}$$

with $${t}_{3}=4{\chi }^{2}\mathrm{[2}{g}^{2}-({\omega }_{b}-i{\gamma }_{q}\mathrm{)(2}{\omega }_{q}-i{\gamma }_{q})]$$, $${t}_{2}=-4i\chi ({\gamma }_{q}+i{{\rm{\Delta }}}_{a}\mathrm{)[2}{g}^{2}-({\omega }_{b}-i{\gamma }_{q}\mathrm{)(2}{\omega }_{q}-i{\gamma }_{q})]$$, $${t}_{1}=\mathrm{[2}{g}^{2}-$$ $$({\omega }_{b}-i{\gamma }_{q}\mathrm{)(2}{\omega }_{q}-i{\gamma }_{q})]({\gamma }_{q}+i{{\rm{\Delta }}}_{a}{)}^{2}$$, $${t}_{0}=\chi {{\rm{\Omega }}}^{2}\mathrm{(2}{\omega }_{q}-i{\gamma }_{q})$$,

13 $${Z}_{0}=\frac{-({\gamma }_{q}^{2}+4{\omega }_{q}^{2})}{({\gamma }_{q}^{2}+4{\omega }_{q}^{2}+8{g}^{2}|{B}_{0}{|}^{2})},$$

14 $${A}_{0}=\frac{{\rm{\Omega }}}{[{\gamma }_{a}+i{{\rm{\Delta }}}_{a}-i\chi ({B}_{0}+{B}_{0}^{\ast })]},$$

15 $${L}_{0}=\frac{2g{B}_{0}{Z}_{0}}{\mathrm{(2}{\omega }_{q}-i{\gamma }_{q})}\mathrm{.}$$

To describe the process of the second-order sideband generation, we define

16 $$\eta =|-2{{\gamma }}_{a}{A}_{2}^{-}/{\varepsilon }|,$$

which is dimensionless, as the efficiency of the second-order sideband generation, where the ${A}_{2}^{-}$ can be obtained from Eq. (18) by analytic solutions in the following method.

### The reason of the appearance of the two-color second-order sideband generation in this hybrid optomechanical system

**Figure 2:**
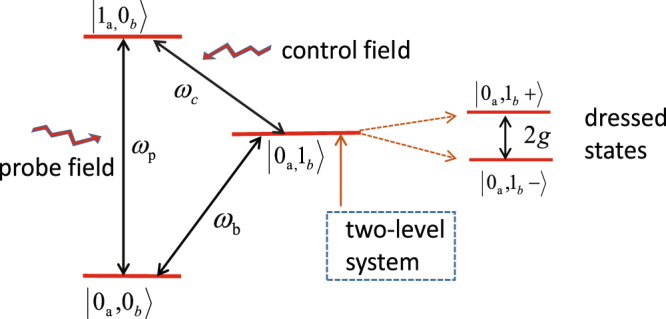
Schematic diagram of the transmission of the photon with the input fields applied to the hybrid system. The single-photon and single-phonon excitations are used to illustrate the photon’s transmission. A typical Λ-type three-level systems consists of three states |0_*a*_, 0_*b*_〉, |0_*a*_, 1_*b*_〉, and |1_*a*_, 0_*b*_〉, where the subscripts a and b represent the photon and phonon states, respectively. However, the state |0_*a*_, 1_*b*_〉 is split into two dressed states |0_*a*_, 1_*b*_+〉 and |0_*a*_, 1_*b*_−〉 when a two-level system is resonantly coupled to the mechanical resonator.

The physical interpretation of the OMIT effect^42^ is not applied to expound the appearance of two transparent windows in the hybrid system where the mechanical resonator is coupled to a two-level system. For the sake of comprehending the physical processes of two transparent windows and two-color second-order sidebands, for simplicity, the single-photon and single-phonon excitations are taken advantage of exemplifying the transmission of photons in Fig. 2 which is similar as in ref.^34^. A typical Λ-type three-level systems is composed of three states |0_*a*_, 0_*b*_〉, |0_*a*_, 1_*b*_〉, and |1_*a*_, 0_*b*_〉, where the subscripts a and b represent the photon and phonon states, respectively. However, the state |0_*a*_, 1_*b*_〉 is split into two dressed states^43^ |0_*a*_, 1_*b*_+〉 and |0_*a*_, 1_*b*_−〉 when the mechanical resonator is resonantly coupled to a two-level system, where the effective resonant frequency of the mechanical resonator becomes *ω*_*b*_ − *g* and *ω*_*b*_ + *g* from *ω*_*b*_ which leads to two transparent windows due to the resonant matching between optical beat frequency Δ and the effective mechanical frequency. Here we consider the higher order upconverted processes^42^ of the control field that the control field absorbs two phonon or the anti-Stokes field absorbs a phonon to generate a second-order sideband which also exhibits two interference windows.

**Figure 3:**
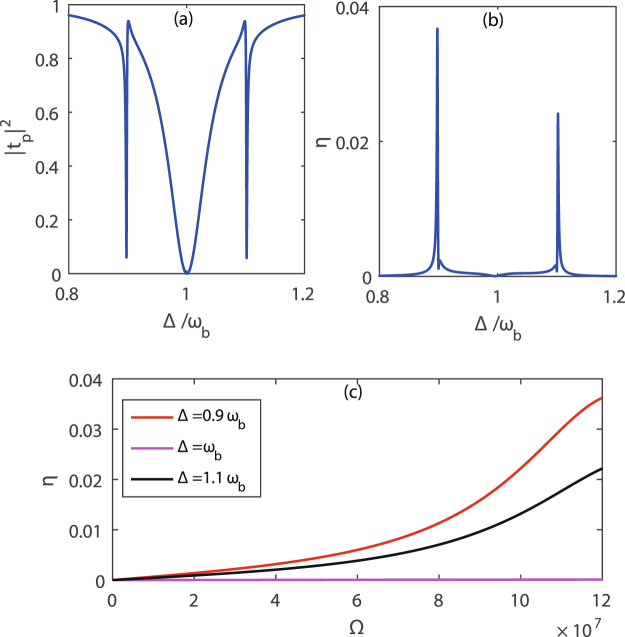
The probe field transmission and the efficiency of the second-order sideband generation. Calculation results of |*t*_*p*_|^2^ and *η* vary with Δ for |Ω|/2*π*  = 19.8 MHz in panels (**a**) and (**b**). *η* is as an function of the control field strength at three valley points Δ = 0.9*ω*_*b*_, *ω*_*b*_ and 1.1*ω*_*b*_ in panel (**c**), assuming *g*/2*π* = 10 MHz, *γ*_*a*_/2*π* = 4 MHz, *ω*_*q*_/2*π* = *ω*_*q*_/2*π* = Δ_*a*_/2*π* = 100 MHz, *γ*_*q*_/2*π* = 0.1 MHz, *γ*_*b*_/2*π* = 1000 Hz, *χ*/2*π* = 10 MHz.

Figure 3(a) and (b) show |*t*_*p*_|^2^ and *η* vary with Δ by solving Eq. (18) and the defined expressions of *t*_*p*_ and *η*. As clearly seen from Fig. 3(a), the value of the |*t*_*p*_|^2^ is very small at Δ = 0.9*ω*_*b*_, *ω*_*b*_ and 1.1*ω*_*b*_ where the probe field is almost completely absorbed and two transparent windows arise. At Δ = *ω*_*b*_, the input laser fields including the control field and the probe field are no longer resonant with the mechanical resonator, because the effective resonant frequencies of the mechanical resonator are *ω*_*b*_ ± *g* after it is coupled to a two-level system. Even though Δ_*a*_ = *ω*_*b*_, the condition, under which a transparent window^42^ appears, can not be satisfied and the cavity field resonantly absorbs the probe field leading to a valley of the |*t*_*p*_|^2^. At Δ = 0.9*ω*_*b*_, or 1.1*ω*_*b*_, whose frequency interval is equal to the splitting width 2*g*, the value of the |*t*_*p*_|^2^ also reaches a valley arising from the Jaynes-Cummings coupling between the mechanical resonator and the two-level system. When Δ = 0.9*ω*_*b*_ or 1.1*ω*_*b*_, nevertheless the Δ_*a*_ is no more equivalent to the effective resonant frequencies *ω*_*b*_ ± *g* of the mechanical resonator, the condition that a transparent window can occur suffers destory, so the probe field is not reflected by the cavity but almost completely absorbed by the mechanical resonator, leading to |*t*_*p*_|^2^ reaching its minimum. The three valleys of the |*t*_*p*_|^2^ at Δ = 0.9*ω*_*b*_, *ω*_*b*_ and 1.1*ω*_*b*_ enable the formation of two transparent windows.

In Fig. 3(b), we display the efficiency *η* of the second-order sideband generation, where two-color second-order sidebands appear at frequencies Δ = 0.9*ω*_*b*_ and 1.1*ω*_*b*_. Considering the interaction between the mechanical resonator and the two-level system, the single-phonon state is split into two corresponding dressed states with frequencies 0.9*ω*_*b*_ and 1.1*ω*_*b*_. When the beat frequency Δ between the input laser fields is equal to the frequency 0.9*ω*_*b*_ or 1.1*ω*_*b*_ of the mechanical resonator, the mechanical resonator starts to oscillate coherently result in many anti-Stokes fields producing. Due to the condition of the OMIT effect ungratified, the anti-Stokes field further absorbs a phonon leading to the second-order sideband generation. However, for Δ = *ω*_*b*_ = Δ_*a*_, the probe field is almost completely absorbed by the cavity field resonance, which is not propitious to second-order sideband generation.

Figure 3(c) shows the efficiency *η* of the second-order sideband generation varies with the control field strength Ω for Δ = 0.9*ω*_*b*_, *ω*_*b*_ and 1.1*ω*_*b*_, respectively. The efficiency *η* of the second-order sideband generation is always monotonous increase with the control field strength Ω for Δ = 0.9*ω*_*b*_ or 1.1*ω*_*b*_. This process is different from the change of the efficiency *η* of second-order sideband generation in the previously mentioned optomechanical system^42^, where the value of *η* enhances when the control field strength is small, and with the control field strength increasing to some degree, the value of *η* begins to decrease. Thanks to the mechanical resonator coupled to a two-level system in the hybrid system, the resonant matching between the detuning Δ_*a*_ of the cavity field and the effective mechanical frequency suffers damage, leading to the probe field being absorbed by the mechanical resonator at Δ = 0.9*ω*_*b*_ or 1.1*ω*_*b*_ with the control field strength Ω increasing. This is advantage to produce a lot of anti-Stokes fields, which farther absorb a phonon to form a second-order sideband, and the efficiency *η* of the second-order sideband generation also gets enhanced. For Δ = *ω*_*b*_, the probe field is resonantly absorbed by the cavity field, which decreases the interaction between the input laser fields and the mechanical resonator, so the efficiency *η* of the second-order sideband generation has hardly changed for different values of the control field strength Ω.

### The features of the two-color second-order sideband generation in this hybrid optomechanical system

**Figure 4:**
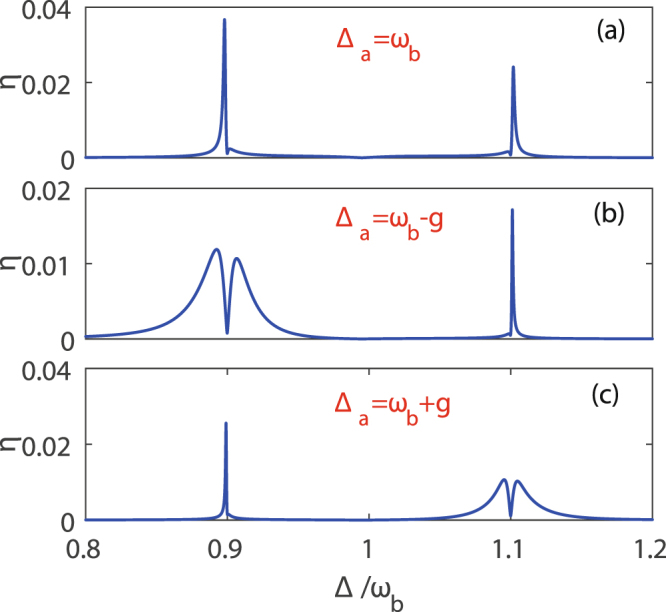
The efficiency *η* of the second-order sideband generation is as a function of Δ for different detunings between the control field and the cavity field. (**a**) Δ_*a*_ = *ω*_*b*_, (**b**) Δ_*a*_ = *ω*_*b*_ − *g* and (**c**) Δ_*a*_ = *ω*_*b*_ + *g* with |Ω|/2*π*  = 19.8 MHz. The other parameters are the same as in Fig. 3.

Based on a general optomechanical system^42^, we take into account that the mechanical resonator is coupled to a two-level system, where the second-order sideband presents some novel features. In Fig. 4, the efficiency *η* of the second-order sideband as a function of Δ for different values Δ_*a*_ is shown. It is found that by transforming the detuning Δ_*a*_ between the control field and the cavity field, the switch between one- and two-color second-order generation can be realized. For Fig. 4(a), two-color second-order sidebands occur when Δ_*a*_ = *ω*_*b*_, which is the same as in Fig. 3(b).

Now we mainly focus on the Fig. 4(b) and (c). When Δ = 0.9*ω*_*b*_ in Fig. 4(b), the efficiency *η* of second-order sideband generation greatly reduces in comparison with in Fig. 4(a), and the value of *η* is close to 0 reaching a local minimum at Δ = 0.9*ω*_*b*_. However at Δ = 1.1*ω*_*b*_, the value of the efficiency *η* of second-order sideband generation reaches a peak. So we can only obtain a obvious second-order sideband in Fig. 4(b). The condition of Δ_*a*_ = 0.9*ω*_*b*_ = Δ in Fig. 4(b) can be used to realize the OMIT effect^42^. Considering the transmission of the probe field under the condition of Δ_*a*_ = 0.9*ω*_*b*_ = Δ, a transparency window can also appear in a general optomechanical system^42^ if the control field strength is strong enough, however the efficiency *η* of the second-order sideband generation gets suppressed. This is why in Fig. 4(b), the value of the efficiency *η* of second-order sideband generation is reduced to 0 at Δ = 0.9*ω*_*b*_ when the control field strength reaches strong enough. The only difference is that the resonant frequency of the OMIT effect is changed from the frequency *ω*_*b*_ of the mechanical resonator to one of effective resonant frequency 0.9*ω*_*b*_ of the mechanical resonator, when the mechanical resonator is coupled to the two-level system. At Δ = 1.1*ω*_*b*_, the probe field is absorbed by the mechanical resonator to generate the anti-Stokes fields and not reflected by the cavity. The anti-Stokes fields in the optical cavity and the control field carry on exciting the mechanical resonator, which is benefited for second-order sideband generation at Δ = 1.1*ω*_*b*_.

Figure 4(c) shows the efficiency *η* of the second-order sideband varies with the Δ for Δ_*a*_ = 1.1*ω*_*b*_. The basic principle in Fig. 4(c), where only a extrusive second-order sideband generates at Δ = 0.9*ω*_*b*_, is the same as in Fig. 4(b). It is worth noting that the position of the second-order sideband generation exchanges between two effective resonant frequencies of the mechanical resonator, because the resonant frequency among the detuning Δ_*a*_ of the cavity field, the beat frequency Δ of the input laser fields and the effective frequency of the mechanical resonator has changed from 0.9*ω*_*b*_ in Fig. 4(b) to 1.1*ω*_*b*_ in Fig. 4(c). In the light of above analysis, we can reach a conclusion that detuning Δ_*a*_ between the cavity field and the control field is an operable variable to realize the switch between the one- and two-color second-order sideband generation.

**Figure 5:**
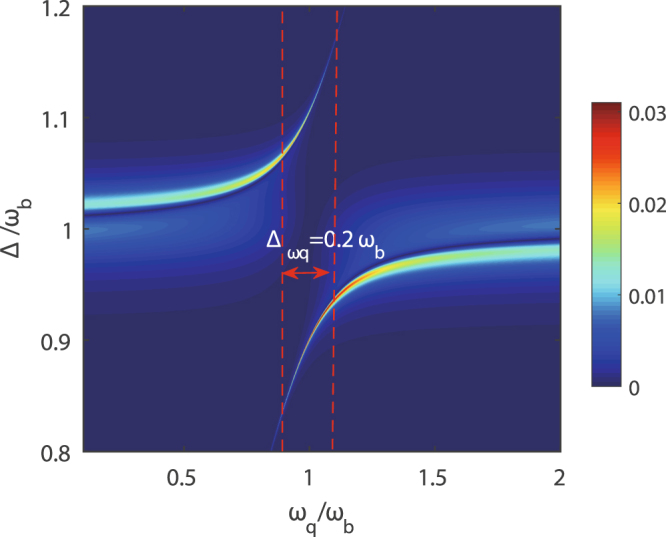
The efficiency *η* of the second-order sideband generation is as a function of Δ and the transition frequency *ω*_*q*_ of the two-level system. |Ω|/2*π*  = 19.8 MHz and the other parameters are the same as in Fig. 3.

In what follows, it is shown that not only the above detuning Δ_*a*_ enables the switch between the one- and two-color second-order sideband generation but also the transition frequency *ω*_*q*_ of the two-level system is an important parameter for whether the two-color second-order sidebands generate. In Fig. 5, the efficiency *η* of the second-order sideband generation is plotted as a function of the detuning Δ between the probe and control fields and the transition frequency *ω*_*q*_ of the two-level system.

Firstly, when we observe the case that the detuning between the transition frequency *ω*_*q*_ of the two-level system and the frequency *ω*_*b*_ of the mechanical resonator is larger than the coupling strength *g*, such as *ω*_*q*_ = 0.5*ω*_*b*_ = 5g or *ω*_*q*_ = 1.5*ω*_*b*_ = 5g, and the value of Δ approaches the frequency *ω*_*b*_ of the mechanical resonator, only a second-order sideband generates. Because if the detuning $|{\omega }_{q}-{\omega }_{b}|\gg g$, the coupling between the mechanical resonator and the two-level system does not work, so the transmission of photons remains unaffected although the mechanical resonator is coupled to a two-level system. It is found that the appearance of two-color second-order sidebands is under the condition that the transition frequency *ω*_*q*_ of the two-level system is (near) resonant with the frequency *ω*_*q*_ of the mechanical resonator, viz. $|{\omega }_{q}-{\omega }_{b}|\le g$. Only the two-level system is resonantly coupled to the mechanical resonator, can the two-color second-order sidebands occur.

In Fig. 5, the second-order sideband generation arises from the directly two-phonon upconverted process of the control field and one-phonon upconverted process of the first-order sideband (anti-Stokes field). For the up conversion of the frequencies, the efficiency *η* of the second-order sideband generation is very low with maximum 3%. If we consider the Stark field from the down converted process of the control field exciting the mechanical resonator, the Stark sideband further absorbs a phonon to generate a second-order sideband, whose efficiency is higher. In general, the down converted process of the frequencies is easier than the up converted process of the frequencies. As shown in Fig. 4(a), we observe that the efficiency *η* of second-order sideband at Δ = 0.9*ω*_*b*_ is higher than the efficiency *η* of second-order sideband at Δ = 0.9*ω*_*b*_. Regardless of the control field and the first-order sideband going through the up or down converted process to generate second-order sidebands, the condition that the two-level system is resonantly coupled to the mechanical resonator must be satisfied for the appearance of two-color second-order sidebands. Therefore, there is another variable the transition frequency *ω*_*q*_ of the two-level system utilized to switch the one- and two-color second-order sideband generation.

**Figure 6:**
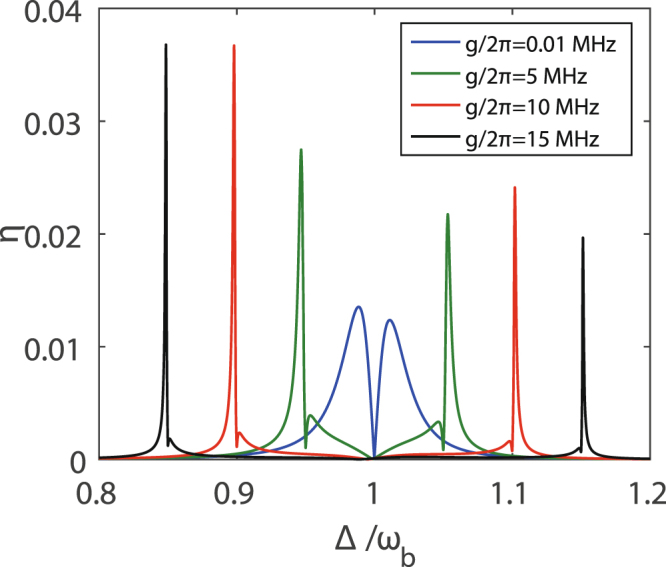
The efficiency *η* of the second-order sideband generation varies with the Δ for different values of the coupling strength *g* between the mechanical resonator and the two-level system. |Ω|/2*π*  = 19.8 MHz and the other parameters are the same as in Fig. 3.

In order to further verify the role of the coupling strength g between the two-level system and the mechanical resonator in the appearance of the two-color second-order sideband generation, Fig. 6 shows the efficiency *η* of the second-order sideband generation for different values of the coupling strength g. The blue curve shows the value of the *η* reaches its local minimum within a frequency window corresponding to about the line width of the cavity at Δ = *ω*_*b*_, which can also take place when the mechanical resonator is not coupled to a two-level system in an optomechanical system^42^. In fact, if the strength of the control field reduces appropriately, and the strength of the control field does not reach the condition of the appearance of OMIT, only a second-order sideband generates at Δ = *ω*_*b*_.

For other curves in Fig. 6, two-color second-order sidebands can be obtained when the two-level system is resonantly coupled to the mechanical resonator for relatively large coupling strength between the mechanical resonator and the two-level system. The splitting width of these two-color second-order sidebands is equal to 2 *g* that results from the Jaynes-Cummings coupling between the mechanical mode and the two-level system. That is to say, by transforming the coupling strength *g* between the mechanical mode and the two-level system, we can get two-color second-order sidebands at different frequencies of the output spectra. In addition, the different values of the coupling strength *g* also have an effect on the efficiency *η* of two-color second-order sideband generation. For example, the efficiency *η* of two-color second-order sideband generation at the coupling strength *g*/*2π* = 10 MHz is the highest, which illustrates the coupling strength should be moderate.

## Discussion

In summary, we have expounded the second-order sideband generation in a hybrid system, consisting of an optical cavity and a mechanical resonator coupled to a two-level system via Jaynes-Cummings interaction. We give the explanation to two-color second-order sideband generation distinguished from one-color second-order sideband generation in the physical mechanism. It can be seen how to achieve the switch between one- and two-color second-order sideband generation by shifting the transition frequency *ω*_*q*_ of the two-level system and the detuning Δ_*a*_ between the cavity field and the control field, whose strength also affects the efficiency *η* of two-color second-order sideband generation. The two-color second-order sidebands at different frequencies of the output spectrum can be observed from the second-order upper sidebands by controlling the coupling parameter *g* between the mechanical resonator and the the two-level system. Our finding of two-color second-order sideband generation maybe be an enlightenment of multi-color second-order sideband generation when the mechanical resonator is coupled to multi-level systems.

It is very difficult for us to get an exactly analytical solution on nonlinear Eqs. (8–11). In the parameter configuration of OMIT, when the control field and the probe field are incident upon the optomechanical system, there are output fields with frequencies *ω*_*c*_ ± *n*Δ where n is positive integer. Instead of consideration on all frequencies of output fields, we are interested in the situation of the second-order sideband generation with *n* = 2. Here we use the perturbation method to deal with the Eqs. (8–11), where the probe field is regarded as a perturbation on the stable system kept by the control field. We assume:

17 $$\begin{array}{rcl}a & = & {A}_{0}+{A}_{1}^{+}{e}^{i{\rm{\Delta }}t}+{A}_{1}^{-}{e}^{-i{\rm{\Delta }}t}+{A}_{2}^{+}{e}^{2i{\rm{\Delta }}t}+{A}_{2}^{-}{e}^{-2i{\rm{\Delta }}t},\\ b & = & {B}_{0}+{B}_{1}^{+}{e}^{i{\rm{\Delta }}t}+{B}_{1}^{-}{e}^{-i{\rm{\Delta }}t}+{B}_{2}^{+}{e}^{2i{\rm{\Delta }}t}+{B}_{2}^{-}{e}^{-2i{\rm{\Delta }}t},\\ {\sigma }_{-} & = & {L}_{0}+{L}_{1}^{+}{e}^{i{\rm{\Delta }}t}+{L}_{1}^{-}{e}^{-i{\rm{\Delta }}t}+{L}_{2}^{+}{e}^{2i{\rm{\Delta }}t}+{L}_{2}^{-}{e}^{-2i{\rm{\Delta }}t},\\ {\sigma }_{z} & = & {Z}_{0}+{Z}_{1}^{+}{e}^{i{\rm{\Delta }}t}+{Z}_{1}^{-}{e}^{-i{\rm{\Delta }}t}+{Z}_{2}^{+}{e}^{2i{\rm{\Delta }}t}+{Z}_{2}^{-}{e}^{-2i{\rm{\Delta }}t}\mathrm{.}\end{array}$$

Then, substituting Eq. (17) to Eqs. (8–11), we can obtain sixteen equations, half of which describe the first-order sideband generation and other parts describe the second-order sideband generation. The concrete manifestation of equations are as follows:

$$\begin{array}{rcl}(i{\rm{\Delta }}+{\gamma }_{a}+i{{\rm{\Delta }}}_{a}){A}_{1}^{+} & = & i\chi ({A}_{0}{B}_{1}^{-\ast }+{A}_{0}{B}_{1}^{+}+{A}_{1}^{+}{{B}_{0}}^{\ast }+{A}_{1}^{+}{B}_{0}),\\ (-i{\rm{\Delta }}+{\gamma }_{a}+i{{\rm{\Delta }}}_{a}){A}_{1}^{-} & = & i\chi ({A}_{0}{B}_{1}^{+\ast }+{A}_{0}{B}_{1}^{-}+{A}_{1}^{-}{{B}_{0}}^{\ast }+{A}_{1}^{-}{B}_{0})+\varepsilon ,\\ (i{\rm{\Delta }}+{\gamma }_{b}+i{\omega }_{b}){B}_{1}^{+} & = & i\chi ({{A}_{0}}^{\ast }{A}_{1}^{+}+{A}_{1}^{-\,\ast }{A}_{0})-ig{L}_{1}^{+},\\ (-i{\rm{\Delta }}+{\gamma }_{b}+i{\omega }_{b}){B}_{1}^{-} & = & i\chi ({{A}_{0}}^{\ast }{A}_{1}^{-}+{A}_{1}^{+\,\ast }{A}_{0})-ig{L}_{1}^{-},\\ (\frac{{\gamma }_{q}}{2}+i{\omega }_{q}+i{\rm{\Delta }}){L}_{1}^{+} & = & ig({B}_{0}{Z}_{1}^{+}+{B}_{1}^{+}{Z}_{0}),\\ (\frac{{\gamma }_{q}}{2}+i{\omega }_{q}-i{\rm{\Delta }}){L}_{1}^{-} & = & ig({B}_{0}{Z}_{1}^{-}+{B}_{1}^{-}{Z}_{0}),\end{array}$$

$$\begin{array}{rcl}(i{\rm{\Delta }}+{\gamma }_{q}){Z}_{1}^{+} & = & -2ig({B}_{0}{L}_{1}^{-\ast }+{B}_{1}^{+}{L}_{0}^{\ast }-{B}_{0}^{\ast }{L}_{1}^{+}-{B}_{1}^{-\ast }{L}_{0}),\\ (-i{\rm{\Delta }}+{\gamma }_{q}){Z}_{1}^{-} & = & -2ig({B}_{0}{L}_{1}^{+\ast }+{B}_{1}^{-}{L}_{0}^{\ast }-{B}_{0}^{\ast }{L}_{1}^{-}-{B}_{1}^{+\ast }{L}_{0}),\\ \mathrm{(2}i{\rm{\Delta }}+{\gamma }_{a}+i{{\rm{\Delta }}}_{a}){A}_{2}^{+} & = & i\chi ({A}_{0}{B}_{2}^{-\ast }+{A}_{0}{B}_{2}^{+}+{A}_{1}^{+}{B}_{1}^{-\ast }\\  &  & +{A}_{1}^{+}{B}_{1}^{+}+{A}_{2}^{+}{{B}_{0}}^{\ast }+{A}_{2}^{+}{B}_{0}),\\ (-2i{\rm{\Delta }}+{\gamma }_{a}+i{{\rm{\Delta }}}_{a}){A}_{2}^{-} & = & i\chi ({A}_{0}{B}_{2}^{+\ast }+{A}_{0}{B}_{2}^{-}+{A}_{1}^{-}{B}_{1}^{+\ast }\\  &  & +{A}_{1}^{-}{B}_{1}^{-}+{A}_{2}^{-}{{B}_{0}}^{\ast }+{A}_{2}^{-}{B}_{0}),\\ ({\gamma }_{b}+i{\omega }_{b}+2i{\rm{\Delta }}){B}_{2}^{+} & = & i\chi ({A}_{0}^{\ast }{A}_{2}^{+}+{A}_{1}^{-\ast }{A}_{1}^{+}+{A}_{2}^{-\ast }{A}_{0})-ig{L}_{2}^{+},\end{array}$$

18 $$\begin{array}{rcl}({\gamma }_{b}+i{\omega }_{b}-2i{\rm{\Delta }}){B}_{2}^{-} & = & i\chi ({A}_{0}^{\ast }{A}_{2}^{-}+{A}_{1}^{+\ast }{A}_{1}^{-}+{A}_{2}^{+\ast }{A}_{0})-ig{L}_{2}^{-},\\ \mathrm{(2}i{\rm{\Delta }}+\frac{{\gamma }_{q}}{2}+i{\omega }_{q}){L}_{2}^{+} & = & ig({B}_{0}{Z}_{2}^{+}+{B}_{1}^{+}{Z}_{1}^{+}+{B}_{2}^{+}{Z}_{0}),\\ (-2i{\rm{\Delta }}+\frac{{\gamma }_{q}}{2}+i{\omega }_{q}){L}_{2}^{-} & = & ig({B}_{0}{Z}_{2}^{-}+{B}_{1}^{-}{Z}_{1}^{-}+{B}_{2}^{-}{Z}_{0}),\\ \mathrm{(2}i{\rm{\Delta }}+{\gamma }_{q}){Z}_{2}^{+} & = & -2ig({B}_{0}{L}_{2}^{-\ast }+{B}_{1}^{+}{L}_{1}^{-\ast }+{B}_{2}^{+}{L}_{0}^{\ast }\\  &  & -{B}_{0}^{\ast }{L}_{2}^{+}-{B}_{1}^{-\ast }{L}_{1}^{+}-{B}_{2}^{-\ast }{L}_{0}),\\ (-2i{\rm{\Delta }}+{\gamma }_{q}){Z}_{2}^{-} & = & -2ig({B}_{0}{L}_{2}^{+\ast }+{B}_{1}^{-}{L}_{1}^{+\ast }+{B}_{2}^{-}{L}_{0}^{\ast }\\  &  & -{B}_{0}^{\ast }{L}_{2}^{-}-{B}_{1}^{+\ast }{L}_{1}^{-}-{B}_{2}^{+\ast }{L}_{0}\mathrm{).}\end{array}$$

In particular, it is pointed out that because *σ*_*z*_ is a Hermitian operator, the relation ${Z}_{0}^{\ast }={Z}_{0}$, ${Z}_{1}^{+\ast }={Z}_{1}^{-}$, ${Z}_{2}^{+\ast }={Z}_{2}^{-}$ are applied to Eq. (18) to simplify calculation.

After some derivation, we can obtain the values of ${A}_{1}^{-}$ and ${A}_{2}^{-}$ to describe the features of main output fields. By using the input-output relation:^44^

19 $${S}_{out}+\frac{{\rm{\Omega }}}{\sqrt{2{\gamma }_{a}}}+\frac{\varepsilon }{\sqrt{2{\gamma }_{a}}}{e}^{-i{\rm{\Delta }}t}=\sqrt{2{\gamma }_{a}}a,$$

the output fields can be described as follows:

20 $${S}_{out}={c}_{0}+{c}_{1}^{+}{e}^{i{\rm{\Delta }}t}+{c}_{1}^{-}{e}^{-i{\rm{\Delta }}t}+{c}_{2}^{+}{e}^{2i{\rm{\Delta }}t}+{c}_{2}^{-}{e}^{-2i{\rm{\Delta }}t},$$

where ${c}_{0}=\sqrt{2{\gamma }_{a}}{A}_{0}-{\rm{\Omega }}/\sqrt{2{\gamma }_{a}}$, ${c}_{1}^{+}=\sqrt{2{\gamma }_{a}}{A}_{1}^{+}$, ${c}_{1}^{-}=\sqrt{2{\gamma }_{a}}{A}_{1}^{-}-\varepsilon /\sqrt{2{\gamma }_{a}}$, ${c}_{2}^{+}=\sqrt{2{\gamma }_{a}}{A}_{2}^{+}$, ${c}_{2}^{-}=\sqrt{2{\gamma }_{a}}{A}_{2}^{-}$. The transmission of the probe field is defined as ${t}_{p}=\frac{\sqrt{2{\gamma }_{a}}{c}_{1}^{-}/\varepsilon }{2}-1$ which is used to discuss the phenomenon of OMIT.

The output field with frequencies *ω*_*c*_ ± *2*Δ is part of the output field with frequencies *ω*_*c*_ ± *n*Δ and it has been studied in different optomechanical systems^27,42^. The term of ${c}_{2}^{-}{e}^{-2i\Delta t}$ and ${c}_{2}^{+}{e}^{2i\Delta t}$ individually describes the sideband process of second-order upper and lower. In this paper we primarily discuss about the amplitude of the second-order upper sideband and of course one can use same method to give a discussion on the characteristic of the second-order lower sideband.

## References

[1] Aspelmeyer M, Kippenberg TJ, Marquardt F (2014). Cavity optomechanics. Rev. Mod. Phys..

[2] Xuereb A, Domokos P (2012). Dynamical scattering models in optomechanics: going beyond the ‘coupled cavities’ model. New J. Phys..

[3] Domokos P, Ritsch H (2003). Mechanical effects of light in optical resonators. J. Opt. Soc. Am. B.

[4] Xiong H, Si LG, Wu Y (2017). Precision measurement of electrical charges in an optomechanical system beyond linearized dynamics. Appl. Phys. Lett..

[5] Gavartin E, Verlot P, Kippenberg TJ (2012). A hybrid on-chip optomechanical transducer for ultrasensitive force measurements. Nat. Nanotechnol..

[6] Krause AG, Winger M, Blasius TD, Lin Q, Painter O (2012). A high-resolution microchip optomechanical accelerometer. Nat.Photonics.

[7] Liu ZX, Wang B, Kong C, Si LG, Xiong H, Wu Y (2017). A proposed method to measure weak magnetic field based on a hybrid optomechanical system. Scientific Reports..

[8] Agarwal GS, Huang SM (2010). Electromagnetically induced transparency in mechanical effects of light. Phys. Rev. A..

[9] Jiang C, Liu H, Cui Y, Li X, Chen G, Chen B (2013). Electromagnetically induced transparency and slow light in two-mode optomechanics.Opt. Express.

[10] Xiong H, Huang YM, Wan LL, Wu Y (2016). Vector cavity optomechanics in the parameter configuration of optomechanically induced transparency. Phys. Rev. A..

[11] Safavi-Naeini AH, Gröblacher S, Hill JT, Chan J, Aspelmeyer M, Painter O (2013). Squeezed light from a silicon micromechanical resonator. Nature (London).

[12] Ma J (2015). Optomechanically induced transparency in the presence of an external time-harmonic-driving force. Scientific Reports..

[13] Weis S (2010). Optomechanically induced transparency. Science..

[14] Blencowe MP (2004). Quantum electromechanical systems. Phys. Rep..

[15] Liu YX, Miranowicz A, Gao YB, Bajer J, Sun CP, Nori F (2010). Qubit-induced phonon blockade as a signature of quantum behavior in nanomechanical resonators. Phys. Rev. A.

[16] Didier N, Pugnetti S, Blanter YM, Fazio R (2011). Detecting phonon blockade with photons. Phys. Rev. B.

[17] Okamoto H, Gourgout A, Chang CY, Onomitsu K, Mahboob I, Chang EY, Yamaguchi H (2013). Coherent phonon manipulation in coupled mechanical resonators. Nat. Phys..

[18] Xiong, H., Liu, Z. X. & Wu, Y. Highly sensitive optical sensor for precision measurement of electrical charges based on optomechanically induced difference-sideband generation. *Opt. Lett.* **42**, 3630 (2017). 10.1364/OL.42.003630 28914919

[19] Gan JH, Xiong H, Si LG, Lü XY, Wu Y (2016). Solitons in optomechanical arrays. Opt. Lett..

[20] Yin T-S, Lü XY, Zheng LL, Wang M, Li S, Wu Y (2017). Nonlinear effects in modulated quantum optomechanics. Phys. Rev. A..

[21] Li JH, Li JH, Xiao Q, Wu Y (2016). Giant enhancement of optical high-order sideband generation and their control in a dimer of two cavities with gain and loss. Phys. Rev. A..

[22] Xiong H, Gan JH, Wu Y (2017). Kuznetsov-Ma Soliton Dynamics Based on the Mechanical Effect of Light. Phys. Rev. Lett..

[23] Kong C, Xiong H, Wu Y (2017). Coulomb-interaction-dependent effect of high-order sideband generation in an optomechanical system. Phys. Rev. A.

[24] Cao C (2016). Tunable high-order sideband spectra generation using a photonic molecule optomechanical system. Scientific Reports.

[25] Wang M (2016). Controllable chaos in hybrid electro-optomechanical systems. Scientific Reports..

[26] Ma J (2014). Formation and manipulation of optomechanical chaos via a bichromatic driving. Phys. Rev. A..

[27] Suzuki H, Brown E (2015). Nonlinear dynamics of an optomechanical system with a coherent mechanical pump: Second-order sideband generation. Phys. Rev. A..

[28] Xiong H, Si LG, Lü XY, Wu Y (2016). Optomechanically induced sum sideband generation. Opt. Express..

[29] Xiong H, Fan YW, Yang X, Wu Y (2016). Radiation pressureinduced difference-sideband generation beyond linearized description. Appl. Phys. Lett..

[30] Javed Akram M, Miskeen Khan M, Saif Farhan (2015). Tunable fast and slow light in a hybrid optomechanical system. Phys. Rev. A.

[31] Blais A, Huang R-S, Wallraff A, Girvin SM, Schoelkopf RJ (2004). Cavity quantum electrodynamics for superconducting electrical circuits: An architecture for quantum computation. Phys. Rev. A.

[32] Grabovskij GJ, Peichel T, Lisenfeld J, Weiss G, Ustinov AV (2012). Strain tuning of individual atomic tunneling systems detected by a superconducting qubit. Science..

[33] Pirkkalainen J-M, Cho SU, Li J, Paraoanu GS, Hakonen PJ, Sillanpaa MA (2013). . Hybrid circuit cavity quantum electrodynamics with a micromechanical resonator. Nature (London)..

[34] Wang H, Gu X, Liu YX, Miranowicz A, Nori F (2014). Optomechanical analog of two-color electromagnetically induced transparency: Photon transmission through an optomechanical device with a two-level system. Phys. Rev. A.

[35] Wang H, Gu X, Liu YX, Miranowicz A, Nori F (2015). Tunable photon blockade in a hybrid system consisting of an optomechanical device coupled to a two-level system. Phys. Rev. A.

[36] Wang ZB, Marzlin KP, Sanders BC (2006). Large cross-phase modulation between slow copropagating weak pulses in 87Rb. Phys. Rev. Lett..

[37] Shiau BW, Wu MC, Lin CC, Chen YC (2011). Low-light-level cross-phase modulation with double slow light pulses. Phys. Rev. Lett..

[38] Yang Q, Hou BP, Lai DG (2017). Local modulation of double optomechanically induced transparency and amplification. Opt. Express.

[39] Ma P, Zhang JQ, Xiao Y, Feng M, Zhang Z (2014). Tunable double optomechanically induced transparency in an optomechanical system. Phys. Rev. A..

[40] Li JH, Yu R, Ding Cl, Wang W, Wu Y (2014). Optical-frequency-comb generation and entanglement with low-power optical input in a photonic molecule. Phys. Rev. A..

[41] Burek MJ, Ramos D, Patel P, Frank IW, Lončar M (2013). Nanomechanical resonant structures in single-crystal diamond. Appl.Phys. Lett..

[42] Xiong H, Si LG, Zheng AS, Yang X, Wu Y (2012). Higher-order sidebands in optomechanically induced transparency. Phys. Rev. A..

[43] Orszag, M. Quantum Optics. (Springer, Berlin, 2000).

[44] Walls, D. F. & J.Milburn, G. Quantum Optics. (Springer, Berlin,1994).

